# The impact of value co-creation on consumer citizenship behavior: Based on consumer perspective

**DOI:** 10.3389/fpsyg.2022.1110011

**Published:** 2023-01-26

**Authors:** Jialiang Yang, Jifan Ren, Meihui Zhao, Haoyu Chen, Huihan Wang

**Affiliations:** ^1^School of Economics and Management, Harbin Institute of Technology (Shenzhen), Shenzhen, Guangdong, China; ^2^College of International Business, Shenyang Normal University, Shenyang, Liaoning, China; ^3^College of Government Management, Shenzhen University, Shenzhen, Guangdong, China

**Keywords:** consumer engagement behavior, consumer citizenship behavior, value co-creation, consumer perceived value, livestreaming platforms

## Abstract

This research investigated the value co-creation behaviors in livestreaming platforms and the internal mechanism of perceived value on consumer value co-creation behavior on the short-video platform TikTok. This research selected the Tiktok platform as the research object, and uses structural equation model to analyze consumer data. The results indicated that consumer-perceived value mediates the relationship between consumer engagement and citizenship behaviors. In addition, short-video platforms exhibit significant community attributes, and interactive behavior forms the primary part of consumer engagement that enhances the perceived value. Consumers join short-video platforms to look for communities that interest them. Consumers’ responses generate the perceived value. Thus, it enhances consumers’ intentions to continue using a particular service, which then increases the likelihood of citizenship behavior. This study also found that consumers’ creative behavior in short-video platforms embodies social and functional values. This suggested that consumers of livestreaming and short-video platforms such as TikTok tend to seek social recognition by sharing their opinions or daily lives. The examination of the mediating role of perceived value on the relationship between consumer engagement behavior and citizenship behavior revealed that perceived value significantly mediated the relationship between consumer engagement behavior and citizenship behavior. This provided evidence of the fact that consumers usually actively participate on the platform by uploading content and sharing their creations, with the motivation to generate significant social impact and gain the recognition of others. Firstly, TikTok’s consumers deeply engage on platforms in which their cognitive messages are integrated into the platform through interacting with others, browsing, and creating short videos. Secondly, TikTok provides a channel with high level of interactivity that facilitates social interaction among strangers. Video uploaders and fans develop positive interactive relationships. Thirdly, TikTok viewers may become video providers at some point; the act of co-creation creates economic value for the platform and generates emotional, functional, and social values for its consumers.

## Introduction

Livestreaming platforms are playing an increasingly important role in real life. With the increasing popularity of mobile devices and Internet technologies, livestreaming platforms break the limits of space and time, and meet consumers’ needs for various types of content consumption that evolve from images and texts to videos. Given the low threshold for creation, the fragmented features, and personalized recommendations, livestreaming platforms have been expanding rapidly (Wang and Liyao, 2019). A survey of social media consumers in 11 countries conducted by the Pew Research Center, a U.S. pollster, revealed that an average of 69% of mobile phone consumers take videos in which 47% of them upload these videos on social media platforms. The consumer size of short-video-based social media platforms is growing rapidly worldwide, ranking the third in global social applications in terms of consumer size (Agrawal et al., 2015; Lee and Watkins, 2016; Khan, 2017). In addition, short videos are easy to produce and widely accepted, thus they unleash the potential for consumer-created content. Therefore, short-video-based social media platforms have been regarded as the next most promising sector for content.

Consumers’ value co-creation occurs during the process of utilization of short-video platforms. Consumers create value by producing, posting and watching short videos, giving likes, providing comments, and replying to messages. Specifically, a video uploader produces and uploads a short video on a social media platform. And then, viewers of the video interact with the uploader in various ways, such as giving likes and comments to the video. In this process, the uploader achieves self-expression and interaction and experiences positive emotions. In the meantime, the viewers receive informative value from the video content. Once the viewers follow the uploader, interaction occurs which gives raise to other social opportunities between them. The platform gains consumer stickiness and economic value simultaneously (Wang and Liyao, 2019). Since uploaders and viewers are the consumers of short-video platforms, their interaction can be seen as the co-creation of consumer value. From the perspective of consumer psychology, consumers can perceive a sense of efficacy of psychological ownership, self-identity, and spatial demand in the process of value co-creation, which can enhance consumers’ loyalty to the brand under the effect of consumer engagement (Gao et al., 2022).

Livestreaming platforms have embodied a significant impact worldwide. However, little academic attention was paid to this topic. Besides, the value co-creation mechanism in livestreaming platforms was not extensively investigated. The extant studies mainly examine content co-creation through the lens of value co-creation between companies and consumers, rather than the value co-creation between individuals. For instance, Zhang et al. (2020) recognized the importance of value co-creation in social media, but they mainly focused on that between companies and consumers. Moreover, few studies verified the importance of livestreaming platforms as the digital brand community in value co-creation.

From the perspective of direct consumer engagement in livestreaming as a social practice, both livestreaming platforms and content creation diffuse the scope of practice through their respective social practice paths on one side, allowing changes in how and what consumers practice (Echeverri and Skalen, 2011), it also reinforces the practice model while consumer engages in the livestreaming process (Snyder et al., 2017). As a result, diverse paths of practice are applied within the period in which consumers are engaged, and the implications of consumer engagement in livestreaming practices are fulfilled. This framework demonstrates consumer citizenship behavior that upholds the image of livestreaming platforms (Warde, 2014). Consumer citizenship behavior is a voluntary and discretionary behavior that contributes to the entire service organization (Groth, 2005), it pertains to the acts in which individuals share positive reviews and comments regarding their experiences through online platforms. These creative ideas contribute to improving service processes and experiences (Assiouras et al., 2019). In the context of online channels, consumer citizenship behavior even helps to induce electronic word of mouth (eWOM) and promote consumer-generated content which can then enhance the competitiveness of organizations (Anaza, 2014). As such, on short-video social media platforms where consumer-generated content forms the core elements, consumer citizenship behavior that is highly relevant to consumer engagement is expected to play a vital role.

Based on the aforementioned practical and theoretical background, this study investigates the livestreaming platform TikTok from the perspective of consumers, builds a conceptual model based on relevant literature and practical analysis, and analyzes real consumer data of TikTok using structural equation modeling (SEM). Since little literature focuses on the value co-creation mechanism of livestreaming platforms, this paper intends to provide an essential theoretical contribution to the study of the value co-creation mechanism of consumers and fill the gap in the research on value co-creation theory in the livestreaming economy. From a practical point of view, this study provides valuable suggestions for improving and optimizing the consumer experience of short-video platforms.

## Literature review and research hypotheses

### Value co-creation behavior

Consumer value co-creation behavior refers to consumers’ actual engagement behavior in value co-creation. The focus of value co-creation research is on the means to motivate consumers’ value co-creation behavior. Monetary incentives have received the most academic attention (Mertins and Walter, 2021). Previous research revealed that more monetary incentives provided to consumers’ value creation facilitate more content creation (Sun et al., 2017). However, the behaviorist motivation theory suggests multiple ways to motivate consumer behavior. In addition to the monetary incentives, consumers’ actual needs including their social and functional needs and emotional needs should be considered as well. Moreover, an in-depth analysis of public material and emotional needs to align each individual’s needs with the organization’s goals is needed. In addition, previous studies did not analyze the specific classification of consumer value co-creation behavior or the specific problems in a case-by-case manner.

Consumer value co-creation behavior is generally classified into consumer engagement behavior and consumer citizenship behavior (Yi and Gong, 2013). Consumer engagement behavior refers to the behavior that consumers must take in the process of producing and providing services to align with the company’s expectations of the services. Consumer engagement behavior involves four dimensions: information seeking, information sharing, responsible behavior, and individual interaction (Yi and Gong, 2013). Consumers interact with providers in the service process by seeking and sharing information, this interaction creates value. Consumers can be psychologically satisfied in the engagement process (Dabholkar, 2015), and such a process is a necessary role-based behavior for the success of value co-creation. McColl-Kennedy et al. (2017) suggested that consumer value creation may positively affect consumer well-being.

Consumer citizenship behavior is evolved from organizational citizenship behavior and is characterized by the partial role that consumers act as employees in interactions regarding service production and delivery. Consumer citizenship behavior is defined as behavior that is spontaneously and independently determined by consumers, not based on the service itself (Groth, 2005). Consumer citizenship behavior can be divided into four dimensions: feedback, defense, assistance, and tolerance (Yi and Gong, 2013). Consumers may actively disseminate reputation, behave in a manner with patience, tolerance, and courtesy in service, make reasonable suggestions to improve service, and help other consumers (Revilla-Camacho et al., 2015). Consumer citizenship behavior is an extra-role behavior that is not necessary for value creation, while it is a resource for companies to gain a competitive advantage (Paine and Organ, 2000). Thus, it can be seen that consumer engagement behavior and consumer citizenship behavior are apparently instrumental in value co-creation (Yi and Gong, 2013). However, few studies have specifically explored the intermediate mechanisms of their interactions to further the investigation of consumer value co-creation (Wang and Liyao, 2019).

Given the neglect of different types of value co-creation behaviors in previous studies, this study investigates the consumers’ value co-creation behaviors in the livestreaming industry based on consumer engagement behavior and consumer citizenship behavior. It not only deepens the understanding of consumer value co-creation behavior, but also offers an important theoretical supplement to the research in this area.

### Consumer-perceived value

In traditional management research, especially in marketing domain, scholars have been interested in the objective value of products. Consumer-perceived value has been received little attention in the relevant research for a long time. However, Ganassali and Matysiewicz (2021) suggested that consumer-perceived value presents a significant impact on companies and brands. Consumer-perceived value is a norm in which consumers measure commodity value, it is a subjective and personalized evaluation by consumers based on their emotions toward the quality and delivery of a commodity (Hapsari et al., 2016). Consumer-perceived value is an essential component in companies and has become one of the most important indicators for measuring business performance (Martelo et al., 2013). The perceived value is defined by consumers and depends on the context, time, and place. It is interaction-based and empirical means, emphasizing value on consumption rather than purchase (Meloni and Swinnen, 2021). In the meanwhile, the perceived value presents subjective and personalized characteristics. For this reason, its specific dimensions should be determined from the perspective of the product or the consumer (Holbrook and Hirschman, 1982). Therefore, this paper emphasizes consumer-perceived value, from the view of the subjective nature of short-video content co-creation and individual differences.

Consumer-perceived value comprises several interrelated dimensions that constitute the overall perception of value (Sanchez-Fernandez and Iniesta-Bonillo, 2007). Currently, there are several ways to classify consumer-perceived value. It includes two-dimensional classification as utility value and hedonic value (Chang and Tseng, 2013); three-dimensional classification as functional value, emotional value, and social value (Holbrook and Hirschman, 1982); four-dimensional classification of functional value, emotional value, social value, and perceived cost (Sweeney et al., 1997); and five-dimensional classification of functional value, emotional value, social value, cognitive value, and associative value (Sheth et al., 1991). In terms of the most recognized classification in the current literature on value co-creation theory, consumer-perceived value is categorized into emotional, functional, and social values (Smith and Colgate, 2007).

Although relevant research gradually attaches importance to consumer-perceived value, this paper considers the following shortcomings in previous studies. Firstly, related studies of content co-creation on short-video platforms failed to examine the core concept of consumer-perceived value. Secondly, research on the impact of consumer-perceived value is conducted from a relatively singular perspective. Specifically, related studies did not investigate the relationship between consumer-perceived value from emotional, functional, and social dimensions; consumer engagement; and consumer citizenship behavior. Finally, previous studies neglected consumer-perceived value on consumer behavior that contributes to companies’ long-term developments. From the perspective of enterprises, consumer citizenship behavior is highly conducive to the sustainable operation and healthy development of short-video-based social media platforms. Based on the characteristics of short-video platforms in the value co-creation process, this study divided consumer-perceived value into emotional value, functional value, and social value and addressed the current research questions in the context of consumer engagement behavior and consumer citizenship behavior.

### Consumer engagement behavior and consumer-perceived value

Consumer engagement behavior refers to the consumers’ behavior associated with the production and delivery of services (Greenwald and Conover, 2014) and is critical for platforms to foster and enhance consumer experience (Wang and Lee, 2020). The consumer engagement behavior of short-video platforms can be classified as browsing, interaction, and creation. Specifically, browsing behavior consists of reading text, watching, searching, and saving short videos. Interactive behavior involves giving likes, providing messages, commenting, replying, and reposting. Creative behavior refers to producing and uploading short videos, and participating in activities on platforms (Dai and Gu, 2017). On short-video platforms, a video uploader creates and uploads a video. Once the video is approved, it will then be recommended to viewers. Viewers will present interactive behaviors, including giving likes and comments, viewing the video uploader’s profile, and following the uploader. In this process, the uploader, the platform, and the viewers complete value co-creation (Wang and Liyao, 2019). The created value is consumer-perceived value.

Perceived value is a comprehensive evaluation based on the psychological perception that consumers experience from a commodity or service, examples of the psychological perception can be given as psychological gains and losses, monetary, and time costs (Martelo et al., 2013). Given that perceived value can be classified as emotional, functional, and social (Chang and Dibb, 2012), when using short-video applications, consumers usually perceive two different values simultaneously. For instance, consumers may feel joy, fun, and happiness. Since studies found that consumers’ perceived emotional value leads to higher product loyalty (Yeh et al., 2016), such perceptions are crucial elements that should be focused. Functional value refers to the value provided by the platform as a tool (Lee et al., 2014). The short-video platform is not only a tool for entertainment, but also it acts as an important tool for consumers to get access to information. Functional value co-creation occurs when consumers access and share messages, record their life experiences or information, and learn knowledge or skills.

As for social value on short-video platforms, social value refers to the effectiveness of enhancing consumers’ self-perception in society (Jahn and Kunz, 2012). In the context of short-video platforms, consumers can get acquainted to individuals with the similar values and interests and gain followers. This function fully satisfies consumers’ real-world social needs in the virtual network space, which enables the co-creation of social values. Different video content, behaviors including liking and commenting allow consumers to perceive themselves as being engaged in the co-creation of emotional value, functional value, and social value. For example, videos with content of life skills offer consumers practical support. Videos with a humorous tone and content with dance possess entertainment value. Therefore, it is believed that the core concept of consumer-led value co-creation theory fit the process of consumers’ use of livestreaming platforms. The similar statement provided by Wu and Qijie (2017) revealed that consumers are permanent co-creators of value, and consumer engagement is a prerequisite for value creation. In consumer engagement, consumers can satisfy their own needs and benefit from the social relationships they build (Gummerus et al., 2012).

From the above-mentioned analysis of consumer-perceived value during using short-video platforms, it is believed that consumer-perceived value, including emotional value, functional value, and social value, originates from the engagement behavior during the process of using short-video platforms. Consumers’ engagement behavior can contribute to their perceived value, in which interactive and creative behaviors positively influence the perceived value. Browsing behavior positively affects the perceived functional value (Dai and Gu, 2017). Although related studies fail to directly investigate the relationship between the use of short-video platforms and perceived value, several studies provide an important theoretical basis for the hypotheses in this paper. Wise et al. (2010) suggested that consumers obtain more positive emotions while using short-video platforms. Yan et al. (2019) found that a group effect occurs when consumers’ emotions are affected by short videos on social media platforms. For example, short videos of major public health events often stimulate empathy among consumers, which then leads to social value co-creation. In addition, consumers of short-video platforms gain extra pleasure and satisfaction through self-expression and interaction. Such emotions create a positive emotional bond between consumers and the video, which enhances perceived value (Hsu and Lin, 2016). Since this study classifies short-video platforms’ consumer-perceived value into emotional, functional, and social value, the following hypotheses were proposed accordingly:

*H1a*: Browsing behavior positively influences the enhancement of emotional value.

*H1b*: Interactive behavior positively influences the enhancement of emotional value.

*H1c*: Creative behavior positively influences the enhancement of emotional value.

*H2a*: Browsing behavior positively influences the enhancement of functional value.

*H2b*: Interactive behavior positively influences the enhancement of functional value.

*H2c*: Creative behavior positively influences the enhancement of functional value.

*H3a*: Browsing behavior positively influences the enhancement of social value.

*H3b*: Interactive behavior positively influences the enhancement of social value.

*H3c*: Creative behavior positively influences the enhancement of social value.

### Consumer-perceived value and citizenship behavior

Once consumers are aware that the value gained from the engagement behavior outweighs the costs, they will become highly satisfied, which enhances their citizenship behavior. In this case, consumers may fully demonstrate their expertise and problem-solving skills in the virtual community, and obtain popularity and satisfaction. This can further enhance consumers’ self-efficacy and facilitate active engagement in value co-creation (Nambisan, 2002). In the context of online shopping, the perceived value may have an impact on various consumers’ citizenship behavior through consumer satisfaction and consumer commitment (Jing, 2015). Furthermore, existing studies stated that perceived value such as benefits of learning, hedonism, socialization, and dignity significantly affect consumer citizenship behavior (Zheng et al., 2013; Balaji, 2014). On the basis of these theoretical discussions, this study hypothesized that perceived value positively impacts citizenship behavior. The hypotheses are described below:

*H4a*: Emotional value positively influences the increase of citizenship behavior.

*H4b*: Functional value positively influences the increase of citizenship behavior.

*H4c*: Social value positively influences the increase of citizenship behavior.

### Consumer engagement behavior and consumer citizenship behavior

Consumers act as both consumers and content producers on social media platforms (Van Doorn et al., 2010). The switch between these two roles gives raise to value co-creation, which positively influences consumer behavior, namely citizenship behavior (Fan et al., 2015). Active consumers of short-video platforms may interact intensively with other consumers through producing and viewing lively and interesting content, which, in turn, promotes positive affective experiences (Hollebeek, 2011), useful knowledge, and social recognition of consumers (Yi et al., 2013). In this context, positive experiences are relevant to the emotional, functional, and social value that consumers derive from the platforms (Zhang et al., 2017). The positive social circumstance generated from interactions provides favorable conditions and potential for citizenship behavior, which contribute to facilitating consumer citizenship behavior (Jung and Yoo, 2017). According to the existing research, the perceived value mediates the value co-creation. Since consumer-perceived value is a form of feedback on consumer behavior that can improve consumer citizenship behavior, this study investigated the value co-creation behavior in livestreaming platforms, and regarded perceived value as a mediating variable. Therefore, the following hypotheses were proposed on the basis of consumer-perceived value as a mediating variable:

*H5*: Perceived value mediates the relationship between engagement behavior and citizenship behavior.

*H5a*: Emotional value mediates the relationship between engagement behavior and citizenship behavior.

*H5b*: Functional value mediates the relationship between browsing behavior and citizenship behavior.

*H5c*: Social value mediates the relationship between browsing behavior and citizenship behavior.

*H5d*: Emotional value mediates the relationship between browsing behavior and citizenship behavior.

*H5e*: Functional value mediates the relationship between browsing behavior and citizenship behavior.

*H5f*: Social value mediates the relationship between interactive behavior and citizenship behavior.

*H5g*: Social value mediates the relationship between interactive behavior and citizenship behavior.

*H5h*: Functional value mediates the relationship between creative behavior and citizenship behavior.

*H5i*: Social value mediates the relationship between creative behavior and citizenship behavior.

**Figure 1:**
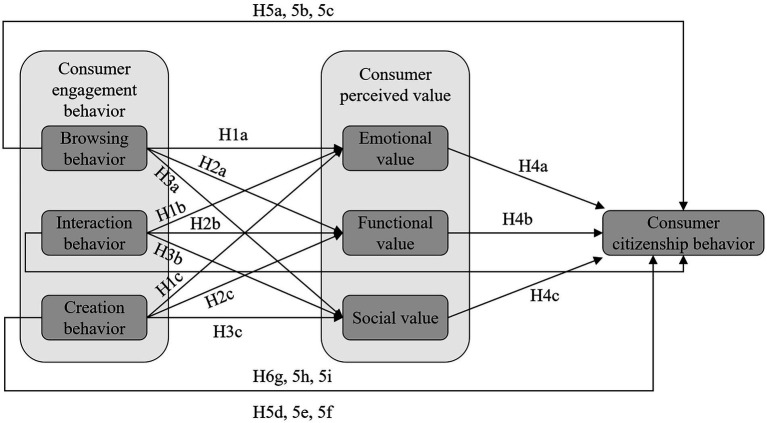
Theoretical model.

Based on the related theories, a mechanism of consumer value co-creation behavior of short-video platforms in the context of livestreaming was built in this study (Figure 1). The model explains the intrinsic relationship between consumer engagement behavior and citizenship behavior, in which the consumer value creation process acts as a mediator.

## Research design

### Data collection

The short-video industry has been growing rapidly in China, the volume of short-video consumers has reached 960 million, which indicates that 7 out of 10 Internet consumers use short-video applications. TikTok, a typical livestreaming platform in short-video communities, has more than 1 billion domestic and international consumers in 2022. Due to the social interaction characteristics of TikTok, the respective consumer behavior is highly representative. As such, consumer behavior on TikTok was chosen as the research object in this study. A questionnaire targeting TikTok consumers was distributed to 1,696 respondents through Wenjuanxing, an online data collection platform. 1,588 questionnaires with valid responses were recovered, indicating a response rate of 93.6%. Table 1 shows the demographic profile of the valid sample.

**Table 1:** Demographic characteristics (*N* = 1,588).

Demographic data		Frequency	Percentage (%)
Gender	Male	739	46.54%
	Female	849	53.46%
Age	Under 18 years old	56	3.53%
	18–30 years old	861	54.22%
	31–40 years old	468	29.47%
	41 years old or above	203	12.78%
Educational background	Undergraduate student	309	19.46%
	Bachelor’s degree	1,115	70.21%
	Master’s degree and above	164	10.33%
Time of use	Within 3 months	300	18.89%
	3–6 months	470	29.6%
	7–12 months	472	29.72%
	12 months or more	346	21.79%

### Scale design

This study explored the value co-creation theory and behaviorist motivation on livestreaming platforms. With the support of previous studies, a questionnaire was designed to meet the actual needs and characteristics of the respondents. Specifically, scales of the questionnaire were designed based on the study of consumer browsing, interaction, and creative behaviors commenced by Dai and Gu (2017; Table 2). The measurement of consumer emotional, functional, and social values was based on the questionnaires conducted by Zhang et al. (2017). Consumer citizenship behavior was measured based on the research by Yi et al. (2013). All items were measured using a 7-point Likert scale (from “strongly disagree” to “strongly agree”).

**Table 2:** Measurement items.

Dimension	No.	Description
Browsing behavior	BB1	I often watch popular short videos recommended on short-video platforms.
	BB2	I often watch full short videos on short-video platforms.
	BB3	I take the initiative to search for short videos that I am interested in on short-video platforms.
	BB4	I often add/save short videos that I think are worth watching on short video platforms to my favorites.
Interactive behavior	IB1	I often give likes to short videos uploaded by others on short-video platforms.
	IB2	I often comment on short videos uploaded by others on short-video platforms.
	IB3	I often reply to other consumers’ comments on short videos I upload on short-video platforms.
Creative behavior	IB4	I often repost short videos uploaded by friends on short-video platforms.
	CB1	I often post my own short-video creations on short-video platforms.
	CB2	I often participate in activities on short-video platforms, such as imitation contests and challenges.
	CB3	I often produce videos using the effects of short-video platforms, such as filters, music, and clips.
Emotional value	EV1	I feel accepted when my friends/followers interact with me.
	EV2	I feel relaxed when I use short-video platforms.
	EV3	I feel delighted when I use short-video platforms.
	EV4	I like to use short-video platforms.
Functional value	FV1	I believe I can share something on short-video platforms.
	FV2	I believe I can acquire something on short-video platforms.
	FV3	I believe I can record something on short-video platforms.
	FV4	I believe I can learn knowledge/skills on short-video platforms.
Social value	SV1	I believe I can meet people/gain more followers on short-video platforms.
	SV2	I believe I can defend my friends/followers on short-video platforms
	SV3	I believe I can increase my reputation and visibility on short-video platforms
	SV4	I believe I can impress others by using short-video platforms
	SV5	I believe I can be recognized by others by using short-video platforms
consumer citizenship behavior	CCB1	I will recommend to my friends or relatives to use short-video platforms.
	CCB2	I will share short videos that interest me through other social media platforms.
	CCB3	I will make positive comments on short-video platforms.
	CCB4	I will tolerate recommended videos that I dislike on short-video platforms.
	CCB5	I will initiate suggestions for improvements on short-video platforms.
	CCB6	I will respond positively to requests from other consumers on short-video platforms.

### Theoretical model and empirical analysis

In this study, structural equation modeling (SEM) was used to examine the research hypotheses. According to the two-step approach, the measurement model was examined to test its reliability and validity before evaluating SEM. SPSS 24.0 was applied to illustrate the population characteristics of the sample and its reliability (Cronbach’s alpha) through descriptive analysis. AMOS 23 was used to verify the validity of each factor, and SEM was performed to test the proposed hypotheses. Maximum likelihood estimation (MLE) was performed to estimate the relevant model parameters. The evaluation criteria of SEM vary; the normalized fit index (NFI) and the root mean square error of approximation (RMSEA) are the indexes generally used by scholars. When NFI is greater than 0.9 and RMSEA is less than 0.05, the model is considered to fit well. If the figure lies between 0.05 and 0.08, it means that the model is acceptable. The critical value of the comparative fit index (CFI) is 0.9 (the higher the better), the critical value of MC is 0.85 (the higher the better), and the critical value of RMSEA is 0.08 (the smaller the better). For the Chi-square-to-freedom ratio, the lower the value, the better the model effect.

### Validity and reliability of the scales

The Cronbach’s alpha for each scale was between 0.809 and 0.887, which exceeded the critical value of 0.70. Therefore, these data were considered as reliable data (Table 3). In addition, the latent variables’ composite reliability (CR) ranged from 0.811 to 0.889, which is greater than the recommended minimum critical value of 0.70. Therefore, the scales used in this study had good internal consistency. The validity measures in this study consist of convergent validity and discriminant validity. Table 2 indicates that the standardized factor loadings for all measured elements ranged from 0.696 to 0.889 which are greater than 0.6. Consequently, all elements were statistically significant. Since the average variance extracted (AVE) values ranged between 0.588 and 0.713 (Table 3), all variables had good convergent validity (Fornell and Larcker, 1981). In addition, the square root of the AVE of the latent variables was greater than the correlation coefficient, specifically generally greater than 0.5 (Table 4). Thus, the adopted scales had fairly excellent discriminant validity.

**Table 3:** Cronbach’s alpha, CR, factor loading, and AVE.

Dimension	No. factor loading		T-value Cronbach’s alpha		CR	AVE
Browsing behavior	BB1	0.825		0.882	0.883	0.655
	BB2	0.741	31.872			
	BB3	0.857	38.092			
	BB4	0.81	35.761			
Interactive behavior	IB1	0.778		0.891	0.85	0.588
	IB2	0.741	28.759			
	IB3	0.834	31.761			
	IB4	0.708	27.429			
Creative behavior	CB1	0.773		0.852	0.854	0.662
	CB2	0.776	30.554			
	CB3	0.886	31.758			
Emotional value	EV1	0.788		0.88	0.882	0.653
	EV2	0.752	31.744			
	EV3	0.758	32.048			
	EV4	0.922	38.749			
Functional value	FV1	0.772		0.893	0.895	0.682
	FV2	0.761	31.604			
	FV3	0.846	35.831			
	FV4	0.916	38.498			
Social value	SV1	0.866		0.902	0.902	0.65
	SV2	0.839	41.752			
	SV3	0.835	41.4			
	SV4	0.741	34.471			
	SV5	0.74	34.392			
Consumer citizenship behavior	CCB1	0.852		0.902	0.903	0.609
	CCB2	0.697	31.186			
	CCB3	0.767	35.704			
	CCB4	0.859	42.414			
	CCB5	0.743	34.086			
	CCC6	0.75	34.56			

CR, composite reliability; AVE, average variance extracted.

**Table 4:** Discriminant validity.

	Consumer citizenship behavior	Social value	Functional value	Emotional value	Creative behavior	Interactive behavior	Browsing behavior
Consumer citizenship behavior	0.780						
Social value	0.607	0.806					
Functional value	0.643	0.604	0.826				
Emotional value	0.525	0.476	0.497	0.808			
Creative behavior	0.477	0.542	0.505	0.234	0.814		
Interactive behavior	0.436	0.524	0.518	0.489	0.408	0.767	
Browsing behavior	0.416	0.321	0.507	0.214	0.421	0.340	0.809

The values on the diagonal are the roots of the dimensional AVE.

### Structural equation model testing

**Figure 2:**
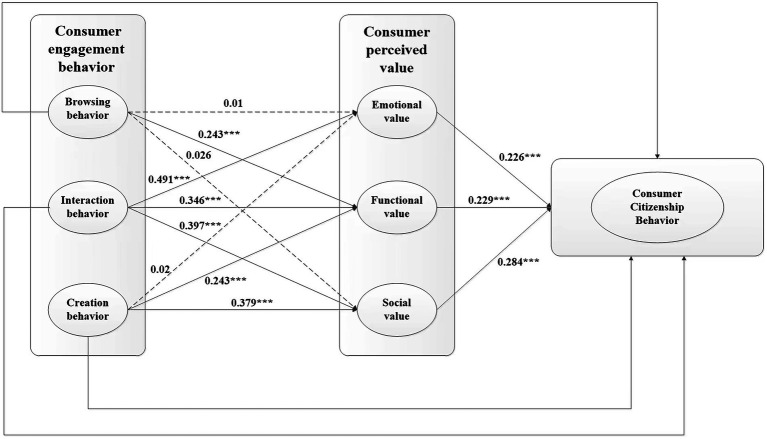
Model results. Significant levels *p < 0.05, **p < 0.01, ***p < 0.001.

The analysis of covariance structures explored the structural relationships between the latent variables, and the results of the fits were as follows. $x^{2}$ =971.947, df = 359, RMSEA = 0.033, GFI = 0.946, AGFI = 0.935, CFI = 0.920, NFI = 0.880, NNFI = 0.913, IFI = 0.921. The figures reached the recommended thresholds, indicating that the fit level of the hypothesized model and the data were acceptable. The results of hypothesis testing are shown in Figure 2 and Table 5. Hypotheses H1b, H2a, H2b, H2c, H3b, H4a, H4b, and H4c were supported for this study. H1a, H1c, and H3a were rejected.

**Table 5:** Results of hypotheses testing.

Hypothesis	Standard load	T-value	*P*	Conclusion
H1a	0.018	0.582	0.561	Rejected
H1b	0.491^***^	14.242	^***^	Supported
H1c	0.024	0.715	0.475	Rejected
H2a	0.243^***^	8.915	^***^	Supported
H2b	0.346^***^	12.027	^***^	Supported
H2c	0.243^***^	8.455	^***^	Supported
H3a	0.026	0.974	0.33	Rejected
H3b	0.397^***^	13.996	^***^	Supported
H3c	0.379^***^	12.967	^***^	Supported
H4a	0.226^***^	7.949	^***^	Supported
H4b	0.229^***^	7.452	^***^	Supported
H4c	0.284^***^	8.942	^***^	Supported

* *p* < 0.05;

** *p* < 0.01;

*** *p* < 0.001.

Consumers’ browsing and creative behaviors do not present significant effect on their emotional value. TikTok consumers’ browsing and creating behaviors tend to emphasize their expression of experiences and works, rather than the interactions with other consumers. This result is consistent with Fredrickson’s psychological theory that emotional value is derived from human contact and interaction. Moreover, browsing behavior had little influence on social value, while creating and interactive behaviors were more important (Frederickson, 1998). Acar et al. (2021) argued that social value represents people’s desire to be recognized and their sense of belonging to society through certain behaviors. However, in browsing behavior among TikTok consumers, consumers merely browsed short videos or watched other consumers’ livestreaming shows without generating direct value to gain recognition from others or society.

### Hypothesis testing

The mediator effect exists once the direct path coefficient between the independent variable and the dependent variable becomes smaller by adding an indirect path to the model (Kuo and Feng, 2013). This study tested nine mediator effects using the bootstrap method, which is consistent with the method applied in the relevant research (Hayes, 2013). Table 6 shows that the estimated functional value for the effect of browsing behavior on citizenship behavior was 0.062, and the 95% confidence intervals of the bias correction approach and the percentile approach did not contain 0. Thus, the mediator effect was valid, and H5b was supported. The estimated emotional value and social value of the effect of browsing behavior on citizenship behavior were 0.003 and 0.011, respectively, and the 95% confidence intervals of the bias correction approach and the percentile approach contained 0. Therefore, the mediator effect was invalid, H5a and H5c were not supported. For the effect of interactive behavior on citizenship behavior, the estimates of emotional value, functional value, and social value were 0.107, 0.080, and 0.120, respectively. The 95% confidence intervals for the bias correction approach and the percentile approach did not contain 0. Therefore, the mediator effect was valid, and the result supported H5d, H5f, and H5e. The estimated functional and social values in the effect of creative behavior on citizenship behavior were 0.063 and 0.119, respectively, and the 95% confidence intervals of the bias correction approach and the percentile approach did not contain 0. Therefore, the mediator effect was valid, and H5h and H5i were supported. The estimate of emotional value in the effect of creative behavior on citizenship behavior was 0.010, and the 95% confidence intervals of the bias correction approach and percentile approach contained 0. Thus, the mediator effect was invalid, and H5g was not supported. The judgments were made on the basis of an analysis conducted by Son et al. (2016) which explored the characteristics of social network sites’ citizenship behavior in terms of consumer value.

**Table 6:** Bootstrap mediator effect test.

Path	Influence coefficient	Standard deviation	Bias corrected (95%)			Percentile (95%)		
			LLCI	ULCI	P	LLCI	ULCI	P
Browsing behavior—emotional value—citizenship behavior	0.003	0.007	−0.011	0.017	0.646	−0.011	0.018	0.637
Browsing behavior—functional value—citizenship behavior	0.062	0.011	0.043	0.087	0.000	0.042	0.085	0.000
Browsing behavior—social value—citizenship behavior	0.011	0.009	−0.006	0.028	0.213	−0.007	0.028	0.222
Interactive behavior—emotional value—citizenship behavior	0.107	0.015	0.078	0.139	0.000	0.078	0.139	0.000
Interactive behavior—functional value—citizenship behavior	0.080	0.013	0.056	0.109	0.000	0.055	0.108	0.000
Interactive behavior—social value—citizenship behavior	0.120	0.017	0.090	0.155	0.000	0.089	0.153	0.000
Creative behavior—emotional value—citizenship behavior	0.010	0.008	−0.003	0.027	0.134	−0.004	0.026	0.159
Creative behavior—functional value—citizenship behavior	0.063	0.011	0.043	0.086	0.000	0.043	0.085	0.000
Creative behavior—social value—citizenship behavior	0.119	0.017	0.088	0.155	0.000	0.087	0.154	0.000

The results showed that the mediating effect of emotional value in the relationship between browsing behavior and citizenship behavior was not significant, as such the effect between creating behavior and consumer citizenship behavior was invalid. As a livestreaming and short-video platform, TikTok is featured with social attributes that allow consumers to communicate and interact with others. However, browsing behavior and creating behavior tend to be consumers’ individual behaviors that do not directly involve communication or interactive activities. In psychological studies, emotional value is considered to be generated by direct contact (Visintin et al., 2017). For this reason, this study innovatively verified that emotional value adequately mediated the relationship between interactive behavior and citizenship behavior. TikTok consumers’ interactive behaviors are considered as sharing and communicating with other consumers, for example, sharing thoughts, works, and feelings among consumers. The emotional value enhanced from these interactions promotes the citizenship behavior of TikTok consumers. However, the path coefficient of the mediator effect of social value between browsing behavior and citizenship behavior was insignificant. It was found that TikTok consumers usually desire to receive likes and comments from others and eventually be recognized by others and society in their field of interest through their short video. However, browsing behavior at this stage failed to generate significant values. This finding differs from the results of the study by Yang et al. (2020) which stated that the pursuit of social value serves as the key driver of consumer citizenship behavior. The rational explanation could be that organizational citizenship behavior was explored under the effect of altruism and observed practice, rather than individual behavior in the context of social networks. The functional value and social value have a significant partial mediating role in the relationship between creative behavior and citizenship behavior. Creating behavior is the most important consumer behavior in short-video platforms, and its core attribute is the community. TikTok consumers create original short videos to promote the platform’s operation and launch community activities to obtain recognition. In this way, consumers fully experience the entertainment and glory associated with TikTok. To sum up, the results of this study indicated that perceived value mediated the relationship between consumer engagement and citizenship behavior, and value creation is the main factor in consumer behavior.

## Conclusion and discussion

### Research conclusion

This study conducted hypothesis testing through structural equation modeling, and drew the following conclusions. Firstly, among the engagement behaviors, only interactive behavior significantly enhances consumers’ perceived emotional value, while browsing behavior and creating behavior have few effects in this regard. Secondly, all three engagement behaviors improve consumers’ perceived functional value of livestreaming platforms. In this case, consumers engage in the value co-creation process on livestreaming platforms by interacting with others, browsing, and creating short videos, and consumers are confident in their ability to share, access, and record the information on livestreaming platforms. Thirdly, browsing behavior fails to contribute to consumers’ perceived functional value as it only occurs at a superficial level of quick message screening, while other interactions, such as creating behaviors, can deepen the perceived social value. Finally, the enhancement of all three perceived values positively affects the consumer citizenship behavior on the platform. Meanwhile, the perceived value has a significant influence as a mediator. However, emotional and social values do not mediate the paths from browsing behavior to consumer citizenship behavior and from interactive behavior to consumer citizenship behavior. Findings are partially aligned with previous studies. The reason could be that this paper explores the mediating effect of perceived value over consumer behavior and engagement, while previous studies usually consider perceived value as an antecedent variable. Additionally, related work by Son et al. (2016) that examined citizenship behavior in social networks merely from the view of social capital theory failed to consider the effect of value from a more comprehensive framework. This paper extends the relevant literature in social media by considering engagement behaviors and consumers’ perceived value from various aspects.

This paper also finds that the creating behavior of consumers in short-video social media platforms tends to be highly associated with social and functional values. Hence, consumers of short-video social media platforms seek social recognition by sharing their views. Functional value and social value among consumers are the two key drivers that promote citizenship behavior. They are also the core values pursued by the consumers of short-video social media platforms. This finding is consistent with the results of Yang et al. (2020) that motivation of social value is the driving factor citizenship behavior. As for the concept of consumer participation behavior, TikTok integrates consumer cognitive information into the sharing on platforms through browsing, interactive, and creative behaviors. Short-video social media platforms offer a channel for a high level of social interaction between video providers and followers. The viewers of TikTok may turn into video providers at some point. The features of TikTok are simple for video providers as it mainly caters to short videos lasting 15 s. However, the entire shooting process is divided into three phases, namely, selecting music, shooting, and post-editing, in which unique elements can be added. Co-creation behavior not only creates economic value for the platform but also generates emotional, functional, and social values for consumers.

## Theoretical contributions and implications

### Theoretical contributions

Firstly, co-creation behavior contributes economic value to the platform and provides emotional, functional, and social value for consumers. Previous studies examined the impact of value co-creation, while they usually consider effect of value co-creation on the contribution to companies’ economic value. However, this paper suggested the social and economic values of co-creation behavior from the consumer’s perspective. As consumers are highly engaged in the short-video platforms, their cognitive messages are integrated into the platforms through their browsing, interactive, and creating behaviors. The related co-creation behavior provides an opportunity for a high level of interactivity which facilitates social interaction among strangers. As a result, consumers of short-video platforms seek social recognition by sharing their opinions or daily lives. In addition, the measurement of the effects of perceived value between consumer engagement behavior and citizenship behavior revealed that perceived value significantly mediated the relationship between consumer engagement behavior and citizenship behavior. This is a new research perspective with significant theoretical value for previous studies.

Secondly, this study examined the internal mechanism of the perceived value between engagement and citizenship behaviors. The results indicated that consumer-perceived value mediated the relationship between consumer engagement and citizenship behaviors. Thus, it contributed to consumers’ intentions to continuously use a particular service and enhances the likelihood of citizenship behavior. Previous studies on value co-creation and the related effects focused on revealing the phenomena, yet they paid less attention to explaining the underlying behavioral mechanisms. This paper highlighted the social value of value co-creation and verifies\d the mediating effect of the perceived value, which provided a new perspective and research framework for studies on the impact of value co-creation.

Thirdly, this paper examined how consumers are involved in developing social practices when they engage in livestreaming shows through the theoretical view of social practices. The value co-creation mechanism was constructed and tested from the perspective of social practice theory through the reinforcement of livestreaming practices by livestreaming platforms (Trowler and Bamber, 2005; Anggraeni et al., 2020) and the proliferation of content creators for their practices (Akaka et al., 2022). Moreover, this paper further revealed the theoretical explanatory power of social practice theory in dissecting the value co-creation process.

### Management implications

The intensity and frequency of TikTok consumers’ daily engagement need to be enhanced in various ways. This study found that consumers’ browsing, interactive, and creating behaviors enhance consumers’ perceptions of emotional, functional, and social value. Furthermore, consumers may engage in citizenship behavior to preserve the platform once their perceived value has been enhanced. Therefore, the enhancement of consumer citizenship behavior needs to be further contextualized according to their engagement behavior. Since engagement and creating behaviors can enhance three perceived values, platforms should place emphasis on developing strategies to simulate positive interactions with consumers and content creators and encourage consumers to create content based on their own experiences. The platform may encourage consumers to browse and repost short videos by setting up reward mechanisms, such as prize draws for reposting. The platform may also plot corresponding short-video contests to directly involve consumers in producing and uploading short videos to increase their perceived value and then, enhance citizenship behavior.

### Research limitations and prospects

It is worthwhile for next studies to further investigate whether there are cross-cultural differences in the findings. Firstly, the research on value co-creation mechanism in live-streaming platforms is still at the early stage, the related literature is scarce. The research on short-video social media platforms as an emerging business model is in its infancy as well. The theoretical basis is relatively limited. Hence, future research in this area needs to be actively promoted. Secondly, although TikTok selected in this study is representative in the field of the short-video social media platforms, differences still exist between distinct live-streaming platforms. Therefore, further discussion of this work should be undertaken. Thirdly, this study proved that perceived value plays an important role between consumer participation and citizenship behavior. However, citizenship behavior may also have an impact on consumer participation. As this paper only conducted one-way research, future work will be done to delve into the co-creation mechanism of live-streaming platforms and the relationship between these important concepts from different perspectives. Related research will be further promoted to different industries to improve the results’ universality and recognition.

## Data availability statement

The raw data presented in this article are not currently available because research based on this data is ongoing. The raw data will be available from the corresponding author upon reasonable request.

## Ethics statement

Ethical review and approval was not required for the study on human participants in accordance with the local legislation and institutional requirements. Written informed consent for participation was not required for this study in accordance with the national legislation and the institutional requirements.

## Author contributions

JY, JR, and MZ conceptualized the study and organized the data collection. JY and JR wrote the first draft of the manuscript. JY and MZ performed the analyses and wrote the results section. HC and HW designed the research framework and made important revisions to the full text. All authors contributed to the article and approved the submitted version.

## Funding

This work was supported by the Postdoctoral research support in Shenzhen (grant number 707-00012444) and Natural Science Foundation of China (grant number 71831005).

## Conflict of interest

The authors declare that the research was conducted in the absence of any commercial or financial relationships that could be construed as a potential conflict of interest.

## Publisher’s note

All claims expressed in this article are solely those of the authors and do not necessarily represent those of their affiliated organizations, or those of the publisher, the editors and the reviewers. Any product that may be evaluated in this article, or claim that may be made by its manufacturer, is not guaranteed or endorsed by the publisher.
